# Collecting core data in severely injured patients using a consensus trauma template: an international multicentre study

**DOI:** 10.1186/cc10485

**Published:** 2011-10-12

**Authors:** Kjetil Gorseth Ringdal, Hans Morten Lossius, J Mary Jones, Jens M Lauritsen, Timothy J Coats, Cameron S Palmer, Rolf Lefering, Stefano Di Bartolomeo, David J Dries, Kjetil Søreide

**Affiliations:** 1 Department of Research, Norwegian Air Ambulance Foundation, Holterveien 24, N-1440 Drøbak, Norway; 2 Division of Emergencies and Critical Care, Oslo University Hospital-Ullevål, Kirkeveien 166, N-0450 Oslo, Norway; 3 Institute of Clinical Medicine, Faculty of Medicine, University of Oslo, Kirkeveien 166, N-0450, Norway; 4 Department of Surgical Sciences, Faculty of Medicine and Dentistry, University of Bergen, N-5021 Bergen, Norway; 5 Mathematics Department, School of Computing and Mathematics, Faculty of Natural Sciences, Colin Reeves Building, Keele University, Keele, Staffordshire ST5 5BG, UK; 6 Orthopaedic Department, Accident Analysis Group, Odense University Hospital, Sdr. Boulevard 29, DK-5000 Odense C, Denmark; 7 Institute of Public Health, Department of Biostatistics, Faculty of Health Sciences, University of Southern Denmark, Campusvej 55, DK-5230 Odense M, Denmark; 8 Emergency Medicine Academic Group, Department of Cardiovascular Sciences, University of Leicester, Infirmary Square, Leicester LE1 5WW, UK; 9 The Trauma Audit & Research Network, Clinical Sciences Building, Hope Hospital, Eccles Old Road, Salford M6 8HD, UK; 10 Trauma Service, The Royal Children's Hospital Melbourne, Flemington Road, Parkville, VIC 3052, Australia; 11 Institute for Research in Operative Medicine, Faculty of Health, University of Witten/Herdecke, Ostmerheimer Str. 200, Haus 38, 51109 Cologne, Germany; 12 Trauma Registry of the German Society of Trauma Surgery, Ostmerheimer Str. 200, 51109 Cologne, Germany; 13 Department of Anaesthesia and ICU, Azienda Ospedaliero-Universitaria di Udine, Piazzale Santa Maria della Misericordia, 33100 Udine, Italy; 14 Italian National Trauma Registry and Emilia-Romagna Trauma Registry, Department of Clinical Governance, Regional Health Agency, Viale Aldo Moro 21, 40127 Bologna, Italy; 15 Department of Surgery, Regions Hospital, 640 Jackson Street, St. Paul, MN 55101, USA; 16 Department of Surgery, University of Minnesota, 420 Delaware Street SE, Minneapolis, MN 55455, USA; 17 Department of Surgery, Stavanger University Hospital, Armauer Hansens vei 20, N-4011 Stavanger, Norway

## Abstract

**Introduction:**

No worldwide, standardised definitions exist for documenting, reporting and comparing data from severely injured trauma patients. This study evaluated the feasibility of collecting the data variables of the international consensus-derived Utstein Trauma Template.

**Methods:**

Trauma centres from three different continents were invited to submit Utstein Trauma Template core data during a defined period, for up to 50 consecutive trauma patients. Directly admitted patients with a New Injury Severity Score (NISS) equal to or above 16 were included. Main outcome variables were data completeness, data differences and data collection difficulty.

**Results:**

Centres from Europe (*n *= 20), North America (*n *= 3) and Australia (*n *= 1) submitted data on 965 patients, of whom 783 were included. Median age was 41 years (interquartile range (IQR) 24 to 60), and 73.1% were male. Median NISS was 27 (IQR 20 to 38), and blunt trauma predominated (91.1%). Of the 36 Utstein variables, 13 (36%) were collected by all participating centres. Eleven (46%) centres applied definitions of the survival outcome variable that were different from those of the template. Seventeen (71%) centres used the recommended version of the Abbreviated Injury Scale (AIS). Three variables (age, gender and AIS) were documented in all patients. Completeness > 80% was achieved for 28 variables, and 20 variables were > 90% complete.

**Conclusions:**

The Utstein Template was feasible across international trauma centres for the majority of its data variables, with the exception of certain physiological and time variables. Major differences were found in the definition of survival and in AIS coding. The current results give a clear indication of the attainability of information and may serve as a stepping-stone towards creation of a European trauma registry.

## Introduction

Major trauma is a leading cause of death and disability around the world [1], and it accounts for approximately 10% of the world's deaths. Globally, unintentional injuries are ranked as the sixth leading cause of death and the fifth leading cause of moderate and severe disability [2]. The introduction of regionalised trauma systems has the potential to reduce preventable deaths [3], but an improved understanding of the benefits and limitations of different trauma care systems requires comparison across systems [4]. However, it has been shown that the datasets of existing trauma registries frequently lack compatible definitions of common data variables [5-9]. Consequently, the comparison and interpretation of trauma system outcomes has been hampered [10]. The lack of dataset uniformity poses substantial challenges to initiatives seeking to assess the quality of healthcare systems [11]. Several regions, particularly in North America, have implemented systematic documentation of trauma care and trauma system performance [12]. However, such documentation is limited in Europe [5,13,14], where no joint trauma registry exists [5,15].

A recent European collaboration (the EuroTARN Group) assessed the potential of creating a data collection trial among a number of trauma registries in Europe, and the potential for comparing summary data and crude mortality rates [5]. Due to differences between trauma registries, the collaboration recognised that meaningful outcome comparisons were not possible. Similar conclusions were also reached in a contemporary Scandinavian report [9].

To address the discrepancies raised in these reports, members of the German, Italian, Scandinavian and UK trauma registries [15,16] performed an expert panel consensus process to develop a core set of uniform patient-level data for documenting and reporting trauma incidents. The resulting template, the Utstein Trauma Template [15-17], consists of recommended eligibility criteria and a set of 36 core data variables with four subsidiary variables.

The aim of the current study was to evaluate the feasibility of collecting patient-level data for severely injured patients across trauma centres using the Utstein Trauma Template variables as a standard.

## Materials and methods

### Study design

The study was a prospective international multicentre feasibility study, in which each participating institution was asked to collect and code up to 50 consecutively hospitalised trauma patients during the study period. The reporting of this study aims at conforming to the STROBE statement for reporting observational studies [18].

### Participants

The primary focus was on inviting trauma registries from a mix of small, medium and large volume European trauma centres. However, to ensure that a degree of valid worldwide comparability was assessed, centres from North America and Australia were also invited.

Trauma centres were invited using a standardised open letter sent by email. For centres that agreed to participate, three reminder emails were sent to those that had not submitted data within the deadline. No follow-up was performed for the institutions that did not respond to the first invitation letter.

### Patients

Trauma centres were asked to include directly and consecutively admitted trauma patients with a New Injury Severity Score (NISS) [19] ≥16 who presented between 1 September 2009 and 30 November 2009. Patients were excluded if they were transferred to the hospital, admitted to the hospital > 24 hours after injury, or if they were declared dead before hospital arrival or with no signs of life upon hospital arrival and no response to initial hospital resuscitation. Patients with asphyxia or drowning injuries and patients who had burns as the predominant injury were also excluded [16].

### Data variables

Participants were asked to collect all the data variables of the Utstein Trauma Template [16] and to fill out and return a self-administered questionnaire (Additional file 1). Using the questionnaire, the centres were asked to report the data variables that they were able to collect, whether their data variable definitions deviated from the definitions of the template, and if they experienced any data collection difficulties. Additional comments could be made for each variable. The centres were asked to grade all injuries according to the Abbreviated Injury Scale (AIS) 2005 or 2005-update 2008 [20], reporting the whole seven digit AIS code.

The main outcome measures were the data completeness of information at the patient and variable levels, the discrepancy for data variable definitions, and the difficulty of data collection. The completeness of the patient-level data was measured on the basis of reported values, while unknown and blank values were considered as missing values. The discrepancy for definitions and the collection difficulty were assessed from the associated questionnaire and were based on "yes", "no" and "unknown" answers.

### Data collection

Patient-level data were collected using the local hospital-based or regional trauma registries. For participants without a suitable registry, an electronic database was provided by the investigators. Centres that did not return the questionnaires, provide the patient-level data, or return a consent form were excluded from the study.

### Study size

The sample size of 50 cases per centre was chosen as a pragmatic size to balance the workload imposed by the study while providing a reasonable number of patients for computing completeness proportions. Low-volume trauma centres that were not able to collect 50 cases within the timeframe were asked to submit three months of hospitalised patients.

### Ethics

The study was approved by the Regional Committee for Medical and Health Research Ethics of Southeast Norway and the Norwegian Social Sciences Data Services. The exported datasets were required to not contain any direct or indirect patient identifiable data. Dates and times were not permitted in the submitted datasets, and patients could not be marked with a reference number that could be linked to a patient number from the submitting centre. The centres were required to return a written and signed consent form stating that the participation and data sharing was in compliance with their own specific institutional and/or national legal frameworks and data protection requirements.

### Statistical methods

Continuous data that were not normally distributed are presented using median and interquartile range (IQR) and analysed using non-parametric techniques. Categorical data are presented as counts and proportions. The completeness of the Utstein core data variables are presented as counts and proportions with 95% confidence intervals (using Wilson's method [21]), and the completeness of each dataset was judged by the number of centres with complete patient data using percentile levels (50^th^, 75^th ^and 100^th^). The desired goal of data completeness was set at ≥80%.

Data were analysed using IBM SPSS version 18 (IBM Company, Chicago, IL, USA) and Stata/SE version 11.1 (StataCorp LP, College Station, TX, USA).

## Results

### Participating centres

**Figure 1:**
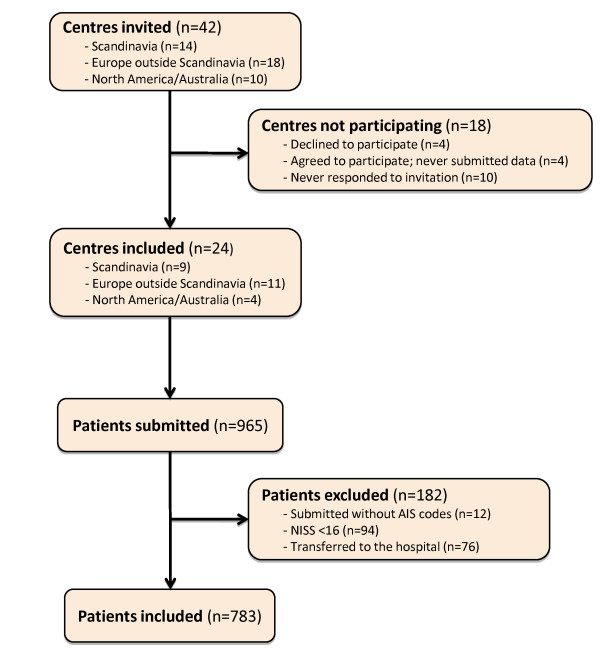
**Flow diagram of invited and included centres and patients**. The number of invited and participating centres, and the number of submitted and included patients are shown.

Of the 42 centres invited, 10 never responded, four centres declined to participate due to resource constraints, and four centres agreed to participate but never submitted the requested material. Twenty-four of the invited centres (57%) participated in the study (Figure 1), of which nine had been part of the Utstein Template development process.

In total, 14 nations were represented. Participation amongst Scandinavian invitees was 64%, participation amongst European invitees outside Scandinavia was 61%, and participation amongst North American/Australian centres was 40%.

Two participants were large, multi-institutional trauma registries that represented collaborations of hospitals (152 and 120 hospitals, respectively), and 20 participants were individual hospitals with a hospital-based registry (Table 1). Two participants did not have a registry prior to the initiation of the study. The two multi-national trauma registries and eight centres with a hospital-based registry had fully or partially implemented the Utstein variables prior to the initiation of the study. The remaining 14 centres only collected the Utstein data for the current study.

**Table 1:** Characteristics of participating centres (*n *= 24)

Centre characteristics	Values
**Data collection for the project, n (%)**	
In own registry	19 (79.2%)
In database designed for purpose	5 (20.8%)
**Utstein data variables implemented prior to study initiation, n (%)**	
Yes	10 (41.7%)
No	14 (58.3%)
**Type of pre-study registry, n (%)**	
Local/regional	20 (83.3%)
National/multi-national	2 (8.3%)
No registry	2 (8.3%)
Number of patients with ISS > 15 received in 2008, median (IQR)	191 (110 to 490); *n *= 17
Number of patients with NISS > 15 in 2008, median (IQR)	200 (78 to 1794); *n *= 10
Number of hospitals the centre/registry represents, median (range)	1 (1 to 152)

IQR, Interquartile range; ISS, Injury Severity Score; NISS, New Injury Severity Score.

Twenty-two of the participating centres submitted data from trauma patients who were consecutively admitted, while the two multicentre trauma registries submitted data from patients who were consecutively included in their trauma registries.

## Patient characteristics

In total, data from 965 patients were submitted. Of these, 182 (19%) were excluded for not meeting the study selection criteria (Figure 1). AIS codes were missing for 12 patients, 94 patients had a NISS < 16, and 76 patients were transferred to the reporting hospital. Therefore, 783 (81%) patients were available for analyses, with 623 (80%) patients from European centres.

Patient characteristics are summarised in Table 2. The majority of the patients were male (73.1%), and the median age was 41.0 years (IQR 24 to 60). Blunt trauma predominated (91.1%), while traffic accidents (53.1%) and high-energy falls (19.3%) were the most prominent injury mechanisms. The median NISS was 27.0 (IQR 20 to 38), and the reported death rate was 14.0%.

**Table 2:** Characteristics of the included trauma patients (*n *= 783)

Demographics	Values
Age, median (IQR)	41 (24 to 60)
**Gender, n (%)**	
Male	572 (73.1%)
Female	211 (26.9%)
**Dominating type of injury, n (%)**	
Blunt	713 (91.1%)
Penetrating	68 (8.7%)
*Missing data*	2 (0.2%)
**Mechanism of injury, n (%)**	
Traffic: motor vehicle accident (excluding motorcycle)	154 (19.7%)
Traffic: motorcycle accident	114 (14.6%)
Traffic: bicycle accident	48 (6.1%)
Traffic: pedestrian	68 (8.7%)
Traffic: other	31 (4.0%)
Shot	36 (4.6%)
Stabbed	31 (4.0%)
Struck or hit by blunt object	33 (4.2%)
Low energy fall	87 (11.1%)
High energy fall	151 (19.3%)
Other	22 (2.8%)
Unknown	5 (0.6%)
*Missing data*	3 (0.4%)
**Injuries grouped by AIS body regions, n (%)**	
Head	1,148 (26.4%)
Face	407 (9.4%)
Neck	16 (0.4%)
Thorax	713 (16.4%)
Abdomen	252 (5.8%)
Spine	426 (9.8%)
Upper extremity	388 (8.9%)
Lower extremity	532 (12.3%)
External and other	85 (2.0%)
*Missing data*	375 (8.6%)
**Injuries grouped by AIS severity levels, n (%)**	
AIS 1 to 3	3,554 (81.9%)
AIS 4 to 6	772 (17.8%)
AIS 9 (unknown)	16 (0.3%)
**NISS groups, n (%)**	
16 to 24	313 (40.0%)
25 to 40	283 (36.1%)
41 to 56	123 (15.7%)
57 to 75	64 (8.2%)
**Survival status, n (%)**	
Died	110 (14.0%)
Survived	621 (79.3%)
Unknown	43 (5.5%)
*Missing data*	9 (1.1%)

AIS, Abbreviated Injury Scale; IQR, Interquartile range; NISS, New Injury Severity Score

### Data variables collected by centres

**Figure 2:**
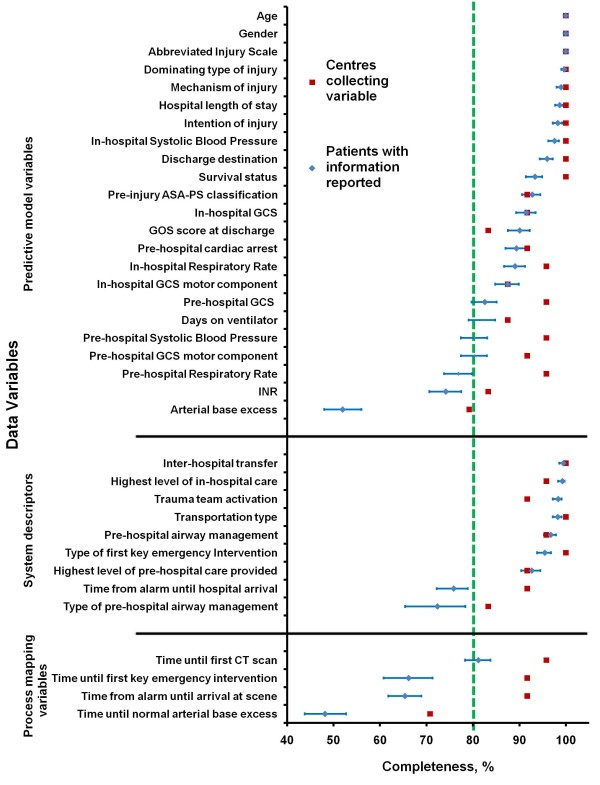
**Completeness of the Utstein core variables among the participating centres**. Current completeness of Utstein core variables (*n *= 24 centres). The proportion of centres collecting each variable, and the proportion of eligible patients with reported information (with 95% CI) are shown.

Of the 36 Utstein variables, 13 (36%) variables were collected in all 24 centres (Table 3). The variable that was recorded by the fewest centres was "Time Until Normal Arterial Base Excess", which was recorded by 17 participants (70.8%) with a completion level of 48.2% (Figure 2 and Additional file 2). Of all the Utstein variables, four (11%) variables did not deviate from the template's definitions in any of the centres (Table 3). Several centres had data variable definitions that differed from the definitions of the Utstein Template. The most heterogeneously defined variable was "Survival Status" (the Utstein recommendation is outcome at Day 30 after injury [11]), and 11 (46%) centres used different definitions (Table 3): six used outcome at end of acute care stay, three used the in-hospital 30-day outcome, and two used the outcome at the end of total somatic stay (including rehabilitation). All centres used the AIS system for anatomical severity scoring. However, only 17 (71%) of the centres used the versions recommended. Two centres submitted the single-digit AIS severity codes, excluding the six-digit injury descriptor.

**Table 3:** Number and proportion of collected Utstein variables, differences in variable definitions, and data collection difficulties

Core data variable	Centres collecting this data variable n (%)	Applied a different definition n (%)	Data variable was difficult to collect n (%)
**Predictive model variables**			
Gender	24 (100%)	0	0
In-hospital SBP	24 (100%)	0	1 (4.2%)
Hospital length of stay	24 (100%)	0	1 (4.2%)
Age	24 (100%)	1 (4.2%)	0
Dominating type	24 (100%)	2 (8.3%)	1 (4.2%)
Intention of injury	24 (100%)	2 (8.3%)	3 (12.5%)
Discharge destination	24 (100%)	4 (16.7%)	2 (8.3%)
Mechanism of injury	24 (100%)	7 (29.2%)	3 (12.5%)
Abbreviated Injury Scale	24 (100%)	8 (33.3%)	1 (4.2%)
Survival status	24 (100%)	11 (45.8%)	4 (16.7%)
In-hospital RR	23 (95.8%)	0	6 (25.0%)
Pre-hospital SBP	23 (95.8%)	1 (4.3%)	5 (21.7%)
Pre-hospital GCS	23 (95.8%)	2 (8.7%)	5 (21.7%)
Pre-hospital RR	23 (95.8%)	2 (8.7%)	8 (34.8%)
Pre-hospital GCS motor component	22 (91.7%)	2 (9.1%)	6 (27.3%)
In-hospital GCS	22 (91.7%)	2 (9.1%)	1 (4.5%)
Pre-hospital cardiac arrest	22 (91.7%)	3 (13.6%)	1 (4.5%)
Pre-injury ASA-PS classification	22 (91.7%)	7 (31.8%)	6 (27.3%)
In-hospital GCS motor component	21 (87.5%)	2 (9.5%)	1 (4.8%)
Days on ventilator	21 (87.5%)	5 (23.8%)	5 (23.8%)
INR	20 (83.3%)	3 (15.0%)	1 (5.0%)
GOS score at discharge	20 (83.3%)	3 (15.0%)	5 (25.0%)
*Pre-hospital SBP-clinical category *^a^	19 (79.2%)^a^	1 (5.3%)^a^	1 (5.3%)^a^
Arterial base excess	19 (79.2%)	1 (5.3%)	3 (15.8%)
*In-hospital RR-clinical category *^a^	18 (75.0%)^a^	2 (11.1%)^a^	2 (11.1%)^a^
*Pre-hospital RR-clinical category *^a^	18 (75.0%)^a^	2 (11.1%)^a^	4 (22.2%)^a^
*In-hospital SBP-clinical category *^a^	17 (70.8%)^a^	1 (5.9%)^a^	1 (5.9%)^a^
**System characteristics**			
Inter-hospital transfer	24 (100%)	1 (4.2%)	2 (8.3%)
Transportation type	24 (100%)	4 (16.7%)	2 (8.3%)
Type of first key emergency intervention	24 (100%)	4 (16.7%)	2 (8.3%)
Highest level of in-hospital care	23 (95.8%)	1 (4.3%)	4 (17.4%)
Pre-hospital airway management	23 (95.8%)	3 (13.0%)	4 (17.4%)
Trauma team activation	22 (91.7%)	2 (9.1%)	3 (13.6%)
Time from alarm until hospital arrival	22 (91.7%)	2 (9.1%)	5 (22.7%)
Highest level of pre-hospital care provided	22 (91.7%)	4 (18.2%)	4 (18.2%)
Type of pre-hospital airway management	20 (83.3%)	4 (20.0%)	4 (20.0%)
**Process mapping data**			
Time until first CT scan	23 (95.8%)	4 (17.4%)	7 (30.4%)
Time from alarm until arrival at scene	22 (91.7%)	1 (4.5%)	6 (27.3%)
Time until first key emergency intervention	22 (91.7%)	4 (18.2%)	4 (18.2%)
Time until normal arterial base excess	17 (70.8%)	2 (11.8%)	6 (35.3%)

^a ^The clinical categories relate to cases for which continuous data were not submitted.

ASA-PS, American Society of Anesthesiologists Physical Status; EMS, emergency medical service; GCS, Glasgow Coma Scale; GOS, Glasgow Outcome Scale; LOS, length of stay; RR, respiratory rate; SBP, systolic blood pressure

Only two variables, "Gender" and "Age", were collected from all centres without difficulty. The variable that was most frequently reported to be difficult to collect was "Pre-Hospital Respiratory Rate", which eight centres (35%) reported as difficult (Table 3).

### Completeness of patient-level core data

The levels of completion for each of the Utstein variables are shown in Figure 2 and Additional file 2. Some centres declined to record specific variables but nevertheless submitted data on those variables for some patients. After exclusion of these datasets, the results showed that 20 Utstein core variables were > 90% complete. Of these, three variables (age, gender, and AIS) were 100% complete. Twenty-eight data variables were > 80% complete. Eight variables had completeness levels that were below the desired 80% threshold (Figure 2). The variables "Time Until Normal Arterial Base Excess", "Arterial Base Excess", and "Pre-Hospital Respiratory Rate", had the lowest levels of completeness.

**Figure 3:**
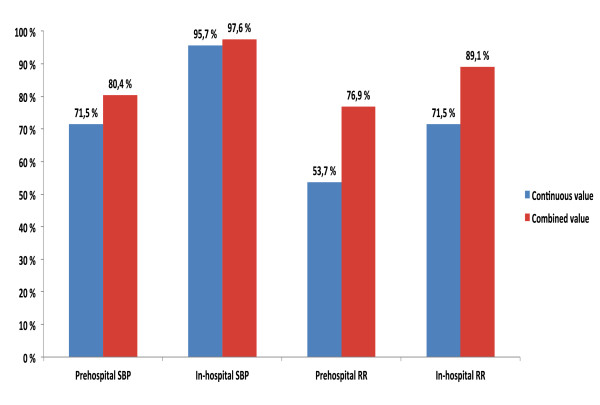
**Completeness of continuous and combined values of systolic blood pressure and respiratory rate**. The figure shows the completeness of continuous values of systolic blood pressure and respiratory rate versus the completeness of a combination of categorical and continuous values of systolic blood pressure and respiratory rate.

For reporting pre- and in-hospital SBP and RR values, the Utstein Template recommends the use of clinical categories (based on the Revised Trauma Score (RTS) categories [22]) when continuous values are missing [15]. This is illustrated in the results presented in Figure 2 and Additional file 2. When the continuous and categorical values of pre-hospital SBP and RR were combined, the completeness increased by 8.9% and 23.2%, respectively (Figure 3). The equivalent in-hospital completeness levels showed an increase of 1.9% and 17.6%.

## Discussion

The current international multicentre study demonstrated acceptable feasibility and completeness in reporting trauma data using a common template. For the majority of variables, the data collection was sufficient, while some areas in need for improvement were identified. The feasibility of bearing this project to fruition may serve as a stepping-stone towards establishment of a common pan-European trauma registry. However, some results deserve further discussion.

This study demonstrated that the data for 28 (78%) of the Utstein variables were > 80% complete, and that the data for 20 (56%) variables were > 90% complete. The pre-hospital SBP and RR values were less complete than were the equivalent in-hospital values. This result is consistent with findings from Arbabi *et al*. [23], who found that pre-hospital and admission SBP values were recorded for 35% and 67% patients, respectively. In cases with missing continuous values, the Utstein Template recommends documenting the SBP and RR values as RTS categories [15,16]. This recommendation is not merely a mathematical consideration; it has a practical sense because clinical categories can be reasonably approximated by palpation of the patient's pulses and by chest examination. In the present study, the combination of the continuous and categorical SBP and RR values resulted in increased completeness compared to the sole use of continuous values (Figure 3). Although categorising continuous data may result in loss of precision and power in addition to other methodological challenges [24,25], the use of the clinical categories provides an undeniable advantage over not having data.

All centres reported injuries according to the AIS system, although injury documentation standards varied. Even though the majority of participating institutions used the AIS dictionaries recommended, nearly 30% did not. Several recent studies have identified differences between the AIS 1998 and 2005/2008 dictionaries in terms of the number of patients classified as 'major trauma' [26-28], illustrating that injury data collected using different AIS dictionaries cannot be directly compared. When comparing outcomes, Injury Severity Score (ISS) [29] or NISS values, AIS dictionary differences could affect the discrimination between severely and less severely injured patients across national and international registries. In light of the recent literature, it is not clear whether parallel coding using the AIS 1998 and AIS 2005/2008 versions should be recommended in order to enable comparisons. However, a solution to overcome the limitation of the existing mapping tool in the AIS dictionary [30] may be a newly developed AIS98 to AIS08 mapping tool [30].

The Utstein Template recommends the use of the short-term outcome variable '30-day survival', which is a mortality indicator that is also applied in other fields such as stroke and acute myocardial infarction [31]. The definitions of the survival outcome variable differed across the participating centres included in the current study. Some centres evaluated short-term outcome based on hospital administrative data, which resulted in the use of in-hospital survival or in-hospital 30-day survival. Others used 30-day survival regardless of whether the patient was still hospitalised. These differences may result in unfavourable biases when trauma care is compared. The use of in-hospital 30-day survival can be particularly problematic with short length of hospital stay or increased tendencies for transfer of patients between facilities. Thus, a greater proportion of deaths within 30 days of injury may be missed if only 'in-hospital deaths' are considered [11,15,31-33]. The endpoints 'in-hospital survival' and '30-day survival' should both be considered included in the Utstein Template until the health care systems have matured to the point where data about 30-day survival status are easily obtainable.

This study does have associated limitations. First, the process applied for identifying centres for invitation was subjective and not standardised. Participating centres may be more likely to comply with the Utstein Template or better able to collect and report data requested. Second, the 10 centres not responding to invitations and the 4 that agreed to participate but never submitted the requested materials were not further contacted. Thus, we cannot preclude the possibility that they found collection of the dataset too difficult or time consuming.

Third, some institutions had already integrated the Trauma Template variables in their trauma documentation protocols and registries prior to the start of the current study, while others only collected these data for the study. Implementation of the template across all centres should have yielded a higher degree of completeness for some data sets. Fourth, participation from North America and Australia was low. However, because of the formalised criteria with which trauma care in American centres is reviewed, there is a greater homogeneity among these centres and data collection. Thus, despite the small number of hospitals, inclusion of three leading centres from the United States gives a good sampling of North American practice. Fifth, the desired goal of completeness (> 80%) used in this study is an arbitrarily chosen threshold. No justifications or guidelines for the acceptability of missing data in registry studies (for example, prognostic studies) exist [personal communication with Professor Douglas G. Altman, University of Oxford, UK]. Thus, the threshold value was a choice based on consensus among the authors. Finally, the template allows some data fields to be left blank when a data variable is unknown or not documented. Leaving a data field blank can make it more difficult to estimate the exact completeness or perform comparative analyses (that is, the exact cause of leaving a data field blank could be "not measured", "forgotten" or "unknown").

This study demonstrates that considerable support exists for the development of an international uniform mandatory core dataset that can be the basis of a European trauma registry. However, several steps still remain. The current Utstein Trauma Template variables and definitions could be further improved before collaborative research on the comparison of trauma care performance is initiated on a larger scale. Hopefully, the results from this study will contribute to improvements. Indeed, at the time of the development of the Utstein Template, the balance between 'desirability' and 'collectability' of a variable was probably in favour of the former because there were no objective data on 'collectability'. This study has identified variables that are particularly difficult to collect. In particular, the collection of "Arterial Base Excess", and process data like "Time Until Normal Arterial Base Excess" needs to be reconsidered or even excluded from the template, while uniform survival outcome variables and type of AIS coding systems used, should be further agreed upon. Additional studies should review the propriety of some of the variables. Furthermore, the data variables should be evaluated with regard to inter-rater reliability [34].

The template was primarily developed for patients who were directly admitted to a trauma centre. A more complete assessment of the performance of the entire trauma system [35] will need to include transferred patients. Exclusion of transferred patients may strongly influence the results when hospitals with large proportions of transferred patients are included.

In order to further develop an international core dataset, a consensus-driven revision of the Utstein Trauma Template, with representatives from multiple continents, should be initiated. The results of the current study will be valuable for such a revision.

## Conclusions

The study shows that 78% of the data variables of the Utstein Trauma Template were > 80% complete. Difficulty with collecting time variables and a lack of uniformity in the use of outcome variables and injury scoring systems across international trauma institutions were found. Overall, the feasibility of collecting most of the core data was demonstrated across several registries and countries.

## Key messages

• The use of the common trauma template was feasible across international registries for the majority of the data variables.

• A lack of uniformity in the use of outcome variables and injury scoring systems across international trauma institutions mandate a need for better standardisation.

• The current results may serve as a stepping-stone towards creation of a European trauma registry.

## Abbreviations

AIS: Abbreviated Injury Scale; IQR: interquartile range; ISS: Injury Severity Score; NISS: New Injury Severity Score; RR: respiratory rate; RTS: Revised Trauma Score; SBP: systolic blood pressure.

## Competing interests

The authors declare that they have no competing interests.

## Authors' contributions

KGR designed the study, prepared and analysed the data, drafted the results, and drafted the manuscript. HML designed the study, assisted in drafting the results, and drafting the manuscript. KS designed the study, assisted in drafting the results, and drafted the manuscript. JMJ designed the study, prepared and analysed the data, assisted in drafting the results, and assisted in drafting the manuscript. JML and CSP assisted in drafting the results, and assisted in drafting the manuscript. TJC, RL, SDP, and DJD assisted in drafting the manuscript. All authors read and approved the final manuscript for publication. The Utstein Trauma Data Collaborators were invited to read and comment on the final manuscript draft.

## Authors' information

KGR, HML, TJC and RL are members of the European Trauma Registry Network's working party for the establishment of a European trauma registry. KGR drafted the Utstein Trauma Template Data Dictionary and is a member of the working party for the establishment of the Norwegian National Trauma Registry. TJC is the chairman of the Trauma Audit and Research Network, UK. CSP chaired the working party of the National Trauma Registry Consortium in Australia and New Zealand for the formulation of a Binational Minimum Dataset (BMDS) and drafted the BMDS Data Dictionary in 2010. RL is co-chairman and statistician of the Trauma Registry of the German Society of Trauma Surgery. SDP is the scientific director of the Italian National Trauma Registry.

## Supplementary Material

Additional file 1 **The self-administered questionnaire**. This file includes the self-administered questionnaire that was distributed to the participating centres. Click here for file

Additional file 2 **Completeness of the Utstein core variables among the participating centres**. The table includes the number of centres collecting a data variable, completeness of patient data from the recording centres, and the number of centres with complete patient data by percentiles. Click here for file
