# Supplementary material for: Collecting core data in severely injured patients using a consensus trauma template: an international multicentre study
Source: Crit Care. 2011 Oct 12;15(5):R237. doi: 10.1186/cc10485 (PMC3334788; doi:10.1186/cc10485)
Supplement: Additional file 1 — The self-administered questionnaire. This file includes the self-administered questionnaire that was distributed to the participating centres. [file cc10485-S1.DOC]

**Additional File 1**

**Questionnaire**

**Utstein Trauma Template: Data implementation status**

1. **Is the AIS 2005 dictionary implemented and used for injury severity coding in the trauma registry?**

🞎 Yes 🞎 No 🞎 Unknown

1. **Which version of the AIS dictionary was used for coding the injury severities of the submitted data?**

🞎 AIS 2005–Update 2008 🞎 AIS 2005 🞎 AIS 1990–Update 1998

🞎 AIS 1990 🞎 Earlier versions 🞎 Other 🞎 Unknown

1. **Does the trauma registry code all injuries a patient has sustained?**

🞎 Yes, all injuries are coded

🞎 No, only three AIS scores, according to the ISS convention, are coded

🞎 No, only three AIS scores, according to the NISS convention, are coded

🞎 No, injuries are coded according to other conventions

1. **If the trauma registry codes injuries according to other conventions, please specify.**

*NB! This is a text field.*

1. **Which database was used for recording the Utstein Template?**

🞎 An institution-based (hospital) trauma registry

🞎 A national trauma registry

🞎 The provided EpiData database

🞎 Other database

1. **If another database was used for recording data, please identify it.**

*NB! This is a text field.*

1. **In this tabular list, please specify whether or not the data variables are currently implemented and recorded, which data have been modified or are different, and whether any data collection difficulties were encountered.**

*NB! When answering these questions, it is important to have the Utstein Trauma Template to hand. Answer the following questions according to each variable definition page in the template.* It is important to describe any modifications to or difficulties with the variables or categories.

| **No.** | **Data variable** | **Was the Utstein template data variable recorded? (Y/N)** | **Is the data variable slightly different from the template or modified? (Y/N)** | **Were there any data variable collection difficulties? (Y/N)** | **Please specify where appropriate: (i) Reason why the data variable was not recorded (ii) Any differences or modifications (iii) Data collection difficulties encountered** |
| --- | --- | --- | --- | --- | --- |
| 1. | Age |  |  |  |  |
| 2. | Gender |  |  |  |  |
| 3. | Dominating type of injury |  |  |  |  |
| 4. | Mechanism of injury |  |  |  |  |
| 5. | Intention of injury |  |  |  |  |
| 6. | Pre-injury ASA-PS Classification |  |  |  |  |
| 7. | Pre-hospital Cardiac Arrest |  |  |  |  |
| 8. | GCS score upon arrival of EMS personnel at scene |  |  |  |  |
| 9. | GCS Motor Component upon arrival of EMS personnel at scene |  |  |  |  |
| 10. | GCS score upon arrival in ED / hospital |  |  |  |  |
| 11. | GCS Motor Component upon arrival in ED / hospital |  |  |  |  |
| 12a. | Syst. Blood Pressure upon arrival of EMS personnel at scene |  |  |  |  |
| 12b. | Syst. Blood Pressure Clinical Category upon arrival of EMS personnel at scene |  |  |  |  |
| 13a. | Syst. Blood Pressure upon arrival in ED / hospital |  |  |  |  |
| 13b. | Syst. Blood Pressure Clinical Category upon arrival in ED / hospital |  |  |  |  |
| 14a. | Resp. Rate upon arrival of EMS personnel at scene |  |  |  |  |
| 14b. | Resp. Rate Clinical Category upon arrival of EMS personnel at scene |  |  |  |  |
| 15a. | Resp. Rate upon arrival in ED / hospital |  |  |  |  |
| 15b. | Resp. Rate Clinical Category upon arrival in ED / hospital |  |  |  |  |
| 16. | Arterial Base Excess |  |  |  |  |
| 17. | Coagulation: INR |  |  |  |  |
| 18. | Number of Days on Ventilator |  |  |  |  |
| 19. | Length of Stay in Reporting Hospital |  |  |  |  |
| 20. | Discharge Destination |  |  |  |  |
| 21. | Glasgow Outcome Scale Score at Discharge from Reporting Hospital |  |  |  |  |
| 22. | Survival Status: 30 day mortality |  |  |  |  |
| 23. | Abbreviated Injury Scale |  |  |  |  |
| 24. | Time from Alarm until Hospital Arrival |  |  |  |  |
| 25. | Highest Level of Pre-Hospital Care Provided |  |  |  |  |
| 26a. | Pre-Hospital Airway Management |  |  |  |  |
| 26b. | Type of Pre-Hospital Airway Management |  |  |  |  |
| 27. | Type of Transportation |  |  |  |  |
| 28. | Type of First Key Emergency Intervention |  |  |  |  |
| 29. | Activation of the Trauma Team |  |  |  |  |
| 30. | Inter-Hospital Transfer |  |  |  |  |
| 31. | Highest Level of In-Hospital Care |  |  |  |  |
| 32. | Time from Alarm until Arrival at Scene |  |  |  |  |
| 33. | Time until Normal Arterial Base Excess |  |  |  |  |
| 34. | Time until First CT Scan |  |  |  |  |
| 35. | Time until First Key Emergency Intervention |  |  |  |  |

1. **Describe any general problems you may have encountered with implementing, documenting, extracting, and/or entering the Utstein data into the trauma registry (i.e., not variable specific problems)?**

*NB! This is a text field.*

1. **Do you have any comments to or further suggestions as to how the Utstein Template Data Dictionary can be improved? Please tell us your ideas in the comments box below.**

*NB! This is a text field.*

**Description of the trauma registry:**

1. **How many trauma patients with ISS>15, were processed (coded and entered) into the trauma registry in 2008?**

__________Patients

1. **How many directly (primary) admitted trauma patients, with ISS>15, were processed (coded and entered) into the trauma registry in 2008?**

__________Patients

1. **How many trauma patients with NISS>15, were processed (coded and entered) into the trauma registry in 2008?**

__________Patients

1. **How many directly (primary) admitted trauma patients, with NISS>15, were processed (coded and entered) into the trauma registry in 2008?**

__________Patients

1. **Does the reporting trauma hospital(s) admit patients younger than 15 years?**

*NB! (WHO definition of pediatric: a child < 15 years of age).*

🞎 Yes 🞎 No 🞎 Unknown

1. **Is the data collector(s) (USA: registrar or data abstractor) who coded the injuries for this project certified by an AAAM-AIS course?**

*AAAM = The Association for the Advancement of Automotive Medicine.*

🞎 Yes 🞎 No, but by an equivalent course 🞎 No

🞎 Unknown

1. **If the data collector(s) is not certified in an AAAM-AIS course, please specify the reason.**

*NB! This is a text field.*

1. **Does the trauma registry have strategies for screening data entry or coding errors, data entry completeness, and cleaning processes?**

🞎 Yes 🞎 No 🞎 Unknown

1. **If the trauma registry has the above-mentioned strategies, please describe the data checking process.**

*NB! This is a text field.*

1. **Which criteria are used for including injured patients in the trauma registry?**

*NB! Multiple answers are accepted (check all that apply).*

🞎 All trauma patients in which the trauma team was prenotified before arrival

🞎 All trauma patients who were met by an activated trauma team

🞎 All patients who sustained one or more traumatic injury

🞎 All trauma patients admitted to a critical care unit

🞎 Trauma patients who were transferred (using ground or air ambulance) IN to the hospital

🞎 Trauma patients who were transferred (using ground or air ambulance) OUT of the hospital to a higher-level acute care hospital

🞎 Trauma patients who were directly admitted to the hospital <24 hours after the injury

🞎 All trauma patients who were admitted (directly or transferred) to the hospital <24 hours after injury

🞎 Trauma patients with an ICD-9 diagnostic code for injuries in the range 800-959.9

🞎 Trauma patients with an ICD-10 diagnostic code for injuries in the range S00–S99, T00–T14, T20–T32, V01–V99, W00–W43, W49–W52, W85–W91, X00–X19, X72–X99, Y00–Y09, and Y22–Y36

🞎 Trauma patients who stayed at the hospital for ≥2 days

🞎 Trauma patients who stayed at the hospital for ≥3 days

🞎 Trauma patients who died in the pre-hospital setting

🞎 Trauma patients who died in the ED

🞎 Trauma patients who died in the hospital

🞎 ISS >15

🞎 NISS >15

🞎 ISS >9

🞎 ISS >10

🞎 Other criteria

🞎 Unknown

1. **If other criteria are used for including injured patients in the trauma registry, please describe the criteria.**

*NB! This is a text field.*

1. **Which criteria are used for excluding injured patients from the trauma registry?**

*NB! Multiple answers are accepted (check all that apply).*

🞎 Elderly patients with fractures of the femoral neck

🞎 Patients who were admitted without a trauma team who have a closed, isolated fracture of one extremity

🞎 Patients with limb fractures

🞎 Trauma patients who were admitted to the hospital >24 hours after injury

🞎 Trauma patients who were transferred in to the hospital

🞎 Patients with superficial injuries

🞎 All trauma patients not admitted to a critical care unit

🞎 Trauma patients admitted to a medical or rehabilitation ward

🞎 Trauma patients whose lengths of stay were <3 days

🞎 Trauma patients with chronic subdural hematoma

🞎 Trauma patients who drowned

🞎 Traumatic asphyxias

🞎 Burn patients if the burn represented the predominant injury or if the patients were treated in a specialized burns unit

🞎 Trauma patients with no signs of life upon hospital arrival and no response to hospital resuscitation

🞎 Trauma patients declared dead before hospital arrival

🞎 Other criteria

🞎 Unknown

1. **If other criteria are used for excluding injured patients from the trauma registry, please describe the criteria.**

*NB! This is a text field.*

1. **How does your trauma registry identify trauma patients considered for inclusion in the trauma registry?**

*NB! Multiple answers are accepted (check all that apply).*

🞎 All trauma patients in which the trauma team were prenotified before arrival are screened

🞎 All trauma patients who were met by an activated trauma team are screened

🞎 All patients admitted to the hospital who sustained one or more traumatic injury are screened

🞎 All trauma patients admitted to a critical care unit are screened

🞎 Trauma patients who were transferred (using ground or air ambulance) IN to the hospital are screened

🞎 Trauma patients who were transferred (using EMS or air ambulance) OUT of the hospital to a higher-level acute care hospital are screened

🞎 Trauma patients who were directly admitted to the hospital <24 hours after the injury are screened

🞎 All trauma patients who were admitted (directly or transferred) to the hospital <24 hours after injury are screened

🞎 Trauma patients with an ICD-9 diagnostic code for injuries in the range 800-959.9 are screened

🞎 Trauma patients with an ICD-10 diagnostic code for injuries in the range S00–S99, T00–T14, T20–T32, V01–V99, W00–W43, W49–W52, W85–W91, X00–X19, X72–X99, Y00–Y09, and Y22–Y36 are screened

🞎 Trauma patients who stayed at the hospital for ≥2 days are screened

🞎 Trauma patients who stayed at the hospital for ≥3 days are screened

🞎 Trauma patients who died in the pre-hospital setting are screened

🞎 Trauma patients who died in the ED are screened

🞎 Trauma patients who died in the hospital are screened

🞎 Other criteria

🞎 Unknown

1. **If other criteria are used for identifying trauma patients, please describe the criteria.**

*NB! This is a text field.*

1. **Which survival outcome variable is currently used in your registry?**

🞎 Outcome at 30 days, regardless of whether the patient was still hospitalized

🞎 Outcome at 30 days, but only if the patient was still hospitalized

🞎 Outcome at end of acute care stay

🞎 Outcome at end of total somatic stay (including rehab)"

🞎 Outcome at six months

🞎 Other (if other please describe) _______________

🞎 Unknown

1. **Which outcome variable was used for this project (please choose from the list below)?**

🞎 Outcome at 30 days, regardless of whether the patient was still hospitalized

🞎 Outcome at 30 days, but only if the patient was still hospitalized

🞎 Outcome at end of acute care stay

🞎 Outcome at end of total somatic stay (including rehab)"

🞎 Outcome at six months

🞎 Other (if other please describe) _______________

🞎 Unknown

**Supplementary Questions**

**Data sharing requirements**

For participation in this study, it is the responsibility of each trauma registry/centre to make sure that participation is in compliance with their respective institutional and/or national legal frameworks and data protection requirements.

In order to define the relevant national legislation and bodies from which the participating hospitals/registries are required to gain permission in order to enable collection and sharing of the Utstein Trauma Template data (Utstein patient dataset), the following two questions should be answered.

1. **Please record if and what legal approvals were required for your registry/hospital to share the Utstein data in the current study?**
2. **If the Utstein data were to be shared on a more permanent/regular basis, would any additional approvals be required?**

🞎 Yes 🞎 No 🞎 Unknown

1. **How many hospitals contribute data to the registry?**

______**Hospital(s)**

**For registries that submit data that were collected in more than one hospital, please answer the following two questions:**

1. **Is the registry able to mandate the quality standards for data collection in each participating hospital?**

🞎 Yes 🞎 No 🞎 Unknown 🞎 Not relevant

1. **Is the quality system (staff training, data checking etc.) the same in all participating hospitals?**

🞎 Yes 🞎 No 🞎 Unknown 🞎 No

**Consent form**

I confirm that participation in the project “International trauma registries’ ability to conform to the revised Utstein Trauma Template” is in compliance with the institutional and/or national legal frameworks and data protection requirements.

----------------------------------------------------------------------------

Trauma Registry / Facility

----------------------------------------------------------------------------

CITY, (STATE) AND COUNTRY

----------------------------------------------------------------------------

DATE

----------------------------------------------------------------------------

Name of responsible Local investigator

----------------------------------------------------------------------------

SIGNATURE OF responsible Local investigator
