# Supplementary material for: Collecting core data in severely injured patients using a consensus trauma template: an international multicentre study
Source: Crit Care. 2011 Oct 12;15(5):R237. doi: 10.1186/cc10485 (PMC3334788; doi:10.1186/cc10485)
Supplement: Additional file 2 — Completeness of the Utstein core variables among the participating centres. The table includes the number of centres collecting a data variable, completeness of patient data from the recording centres, and the number of centres with complete patient data by percentiles. [file cc10485-S2.DOC]

**Additional File 2**

| **Additional Table 2. Completeness of the Utstein core variables among the participating centres.** | | | | | | | | |
| --- | --- | --- | --- | --- | --- | --- | --- | --- |
| **Core data variable** | **Centres collecting this data variable**  n (%) | **Information from centres reporting this variable** | | | | | | |
|  |  | **Completeness of patient data from the recording centres** | | | | **Number of centres with complete patient data by percentiles** | | |
|  |  | **Number of patients available for analyses**  n | **Number of patients with reported information**  n | **Proportion of patients with reported information**  % | **95% CI** | **50^th^** | **75^th^** | **100^th^** |
| **Predictive model variables** | | | | | | | | |
| Age | 24 (100%) | 783 | 783 | 100% | 99.5-100% | 24 | 24 | 24 |
| Gender | 24 (100%) | 783 | 783 | 100% | 99.5-100% | 24 | 24 | 24 |
| Abbreviated Injury Scale | 24 (100%) | 783 | 783 | 100% | 99.5-100% | 24 | 24 | 24 |
| Dominating type of injury | 24 (100%) | 783 | 781 | 99.7% | 99.0-100% | 24 | 24 | 23 |
| Mechanism of injury | 24 (100%) | 783 | 775 | 99.0% | 98.0-99.5% | 24 | 24 | 17 |
| Hospital length of stay | 24 (100%) | 783 | 773 | 98.7% | 97.7-99.3% | 24 | 24 | 22 |
| Intention of injury | 24 (100%) | 783 | 770 | 98.3% | 97.2-99.2% | 24 | 24 | 16 |
| In-hospital SBP | 24 (100%) | 783 | 749 | 95.7% | 94.0-96.9% | 24 | 24 | 12 |
| Discharge destination | 24 (100%) | 783 | 752 | 96.0% | 94.4-97.2% | 24 | 23 | 18 |
| Survival status | 24 (100%) | 783 | 731 | 93.4% | 91.4-94.9% | 23 | 23 | 20 |
| Pre-hospital GCS | 23 (95.8%) | 780 | 644 | 82.6% | 79.7-85.1% | 22 | 18 | 6 |
| In-hospital RR | 23 (95.8%) | 780 | 558 | 71.5% | 68.3-74.6% | 18 | 13 | 6 |
| Pre-hospital SBP | 23 (95.8%) | 780 | 558 | 71.5% | 68.3-74.6% | 22 | 18 | 6 |
| Pre-hospital RR | 23 (95.8%) | 780 | 419 | 53.7% | 50.2-57.2% | 14 | 7 | 2 |
| Pre-injury ASA-PS classification | 22 (91.7%) | 683 | 634 | 92.8% | 90.6-94.5% | 22 | 20 | 10 |
| In-hospital GCS | 22 (91.7%) | 691 | 633 | 91.6% | 89.3-93.5% | 20 | 17 | 13 |
| Pre-hospital GCS motor component | 22 (91.7%) | 754 | 606 | 80.4% | 77.4-83.0% | 21 | 16 | 5 |
| Pre-hospital cardiac arrest | 22 (91.7%) | 736 | 658 | 89.4% | 87.0-91.4% | 21 | 20 | 16 |
| In-hospital GCS motor component | 21 (87.5%) | 665 | 582 | 87.5% | 84.8-89.8% | 18 | 15 | 8 |
| Days on ventilator | 21 (87.5%) | 690 | 567 | 82.2% | 79.1-84.8% | 19 | 15 | 12 |
| GOS score at discharge | 20 (83.3%) | 635 | 572 | 90.1% | 87.5-92.2% | 20 | 18 | 9 |
| INR | 20 (83.3%) | 609 | 452 | 74.2% | 70.6-77.5% | 16 | 13 | 2 |
| Arterial base excess | 19 (79.2%) | 590 | 307 | 52.0% | 48.0-56.0% | 10 | 8 | 2 |
| *Pre-hospital SBP - clinical category* ^a^ | 19 (79.2%) | 570 | 69^a^ | 12.1%^a^ | 9.7-15.0% | 1 | 0 | 0 |
| *Pre-hospital RR - clinical category* ^a^ | 18 (75.0%) | 520 | 181^a^ | 34.8%^a^ | 30.8-39.0% | 4 | 1 | 0 |
| *In-hospital RR - clinical category* ^a^ | 18 (75.0%) | 520 | 137^a^ | 26.3%^a^ | 22.7-30.3% | 2 | 1 | 0 |
| *In-hospital SBP - clinical category* ^a^ | 17 (70.8%) | 475 | 13^a^ | 2.7%^a^ | 1.6-4.6% | 0 | 0 | 0 |
|  |  |  |  |  |  |  |  |  |
| **System characteristics descriptors** | | | | | | | | |
| Inter-hospital transfer | 24 (100%) | 783 | 779 | 99.5% | 98.6-99.9% | 24 | 24 | 23 |
| Transportation type | 24 (100%) | 783 | 770 | 98.3% | 97.2-99.1% | 24 | 24 | 16 |
| Type of first key emergency intervention | 24 (100%) | 783 | 748 | 95.5% | 93.8-96.8% | 23 | 21 | 18 |
| Highest level of in-hospital care | 23 (95.8%) | 694 | 689 | 99.3% | 98.3-99.7% | 22 | 22 | 18 |
| Pre-hospital airway management | 23 (95.8%) | 757 | 733 | 96.8% | 95.3-97.9% | 23 | 23 | 18 |
| Trauma team activation | 22 (91.7%) | 689 | 678 | 98.4% | 97.2-99.1% | 22 | 21 | 18 |
| Time from alarm until hospital arrival | 22 (91.7%) | 730 | 554 | 75.9% | 72.7-78.9% | 18 | 14 | 5 |
| Highest level of pre-hospital care provided | 22 (91.7%) | 644 | 597 | 92.7% | 90.4-94.5% | 22 | 20 | 8 |
| Type of pre-hospital airway management | 20 (83.3%) | 181^b^ | 131^b^ | 72.4% | 65.4-78.4% | 17 | 14 | 14 |
|  |  |  |  |  |  |  |  |  |
| **Process mapping variables** | | | | | | | | |
| Time until first CT scan | 23 (95.8%) | 778 | 632 | 81.2% | 78.3-83.8% | 21 | 18 | 1 |
| Time from alarm until arrival at scene | 22 (91.7%) | 691 | 452 | 65.4% | 61.8-68.9% | 15 | 11 | 2 |
| Time until first key emergency intervention | 22 (91.7%) | 311^c^ | 206^c^ | 66.2% | 60.8-71.3% | 17 | 12 | 5 |
| Time until normal arterial base excess | 17 (70.8%) | 483^d^ | 233^d^ | 48.2% | 43.8-52.7% | 10 | 7 | 3 |
| a: The clinical categories and figures relate to cases for which continuous data were not submitted.  b: Cases from centres that allegedly did not record this data variable (n=211), and patients in which no pre-hospital airway management was performed (n=391), were excluded.  c: Cases from centres that allegedly did not record this variable (n=73), and cases in which no emergency intervention was performed (n=399), were excluded.  d: Cases from centres that allegedly did not record this variable (n=300), were excluded.  ABE: Arterial base excess; ASA-PS: American Society of Anesthesiologists Physical Status Classification System; CI: Confidence interval; EMS: Emergency medical service; GCS: Glasgow Coma Scale; GOS: Glasgow Outcome Scale; LOS: Length of stay; RR: Respiratory rate; SBP: Systolic blood pressure. | | | | | | | | |
